# Phenanthrene exposure induces cardiac hypertrophy via reducing miR-133a expression by DNA methylation

**DOI:** 10.1038/srep20105

**Published:** 2016-02-01

**Authors:** Lixing Huang, Zhihui Xi, Chonggang Wang, Youyu Zhang, Zhibing Yang, Shiqi Zhang, Yixin Chen, Zhenghong Zuo

**Affiliations:** 1 State Key Laboratory of Cellular Stress Biology, School of Life Sciences, Xiamen University, Xiamen 361005, China; 2 State Key Laboratory of Marine Environmental Science, Xiamen University, Xiamen 361005, China; 3 Fisheries College, Key Laboratory of Healthy Mariculture for the East China Sea, Ministry of Agriculture, Jimei University, Xiamen, Fujian 361021, P.R. China

## Abstract

Growing evidence indicates that there is an emerging link between environmental pollution and cardiac hypertrophy, while the mechanism is unclear. The objective of this study was to examine whether phenanthrene (Phe) could cause cardiac hypertrophy, and elucidate the molecular mechanisms involved. We found that: 1) Phe exposure increased the heart weight and cardiomyocyte size of rats; 2) Phe exposure led to enlarged cell size, and increased protein synthesis in H9C2 cells; 3) Phe exposure induced important markers of cardiac hypertrophy, such as atrial natriuretic peptide, B-type natriuretic peptide, and c-Myc in H9C2 cells and rat hearts; 4) Phe exposure perturbed miR-133a, CdC42 and RhoA, which were key regulators of cardiac hypertrophy, in H9C2 cells and rat hearts; 5) Phe exposure induced DNA methyltransferases (DNMTs) in H9C2 cells and rat hearts; 6) Phe exposure led to methylation of CpG sites within the miR-133a locus and reduced miR-133a expression in H9C2 cells; 7) DNMT inhibition and miR-133a overexpression could both alleviate the enlargement of cell size and perturbation of CdC42 and RhoA caused by Phe exposure. These results indicated that Phe could induce cardiomyocyte hypertrophy in the rat and H9C2 cells. The mechanism might involve reducing miR-133a expression by DNA methylation.

Heart diseases kill about 17 million people around the world every year. It is the leading cause of mortality in the United States, responsible for about one of every three deaths^1^, and the percentage of adults with diagnosed heart disease has reached 11.5%^2^. At the same time, cardiovascular deaths and disease have increased at a fast rate in low- and middle-income countries^3^. Hypertrophy frequently progresses to heart failure, arrhythmia, and sudden death, and is the primary cause of mortality worldwide. Hypertrophy also increased sharply over recent decades^4^.

More and more evidence indicates that air pollution is a risk factor for heart diseases^5^,^6^,^7^,^8^,^9^. Some researchers have demonstrated that pollution including PM_2.5_, PM_10,_ traffic air pollution, carbon monoxide, and persistent organic pollutants (including polychlorinated biphenyls, dioxins, and polycyclic aromatic hydrocarbons (PAHs)) can lead to cardiac hypertrophy^10^,^11^,^12^,^13^,^14^,^15^,^16^.

PAHs are ubiquitous environmental contaminants that are primarily generated through anthropogenic activities such as incomplete burning of fossil fuels, oil, wood, and organic matter. Phenanthrene (Phe) is a main component of environmental PAHs and occurs in various ambient sources, i.e. in particulate matter formed from combusted smoky coal^17^, in soil and sediment samples^18^, in diesel emissions^19^ and tobacco smoke^20^, as well as in foodstuffs^21^,^22^,^23^. Phe is also found in the air, and in some water sources. Air concentrations of Phe in London are 76.1–82.0 ng/m^3^, and in the pore water of the Jiulong River Estuary and Western Xiamen Sea they are 2.44–26.1 μg/L (about 10–150 nmol/L)^24^,^25^. Therefore, humans easily contact Phe in their daily life, for example, by diet, breathing, smoking, drinking and occupational exposure. Other research in our laboratory also showed that Phe was one of the most abundant PAHs in the peripheral venous blood of pregnant women (non-smoker) and the cord blood of newborn (37.61 ± 0.47 and 35.32 ± 0.34 μg/L, respectively). Phe is connected to cardiac toxicity in the early life stages of fish embryos, and is manifested as heart failure and arrhythmia^26^,^27^,^28^,^29^, which are usually the pathological end point of cardiac hypertrophy. There is now an emerging link between ambient urban air and human cardiovascular disease, but the primary toxic components of urban air remain elusive. Incardona *et al.*^30^ indicate that Phe in airborne sources is bioavailable and likely to be toxic to the human heart when inhaled, and should be considered a prime suspect in the cardiovascular impacts of urban air.

As we know, cardiac hypertrophy is a common pathophysiological change of cardiac diseases, including hypertension, myocardial infarction and valvulopathy. At the cellular level, cardiac hypertrophy is characterized by enlarged cell size, increased protein synthesis, an increase in intracellular Ca^2+^ concentration, and reactivation of the fetal gene program^31^,^32^, including cardiac hypertrophy markers such as the ANP, BNP, and c-Myc.

Defined miRNAs and transcription factors are involved in the process of cardiac hypertrophy. For example, miR-133a is demonstrated to have a critical role in determining cardiomyocyte hypertrophy by regulating its specific target genes: RhoA, a GDP-GTP exchange protein regulating cardiac hypertrophy; CdC42, a signal transduction kinase implicated in hypertrophy; and Nelf-A/WHSC2, a nuclear factor involved in cardiogenesis^33^.

However, little is known about the mechanism of Phe induced cardiac hypertrophy from earlier studies. Moreover, most of the studies concerning Phe focus on fish, and there are few reports concerning the symptoms and mechanisms of exposure to low dose Phe on mammals.

In the present study, rat and H9C2 rat cardiomyoblast cells exposed to low dose Phe were used to 1) determine whether low dose Phe exposure was capable of inducing cardiac hypertrophy; 2) detect the symptoms caused by Phe exposure *in vivo* and *in vitro*; and 3) gain an insight into the mechanism(s) underlying low dose Phe-induced cardiac hypertrophy.

## Results

### Phe induced cardiac hypertrophy *in vivo*

**Figure 1:**
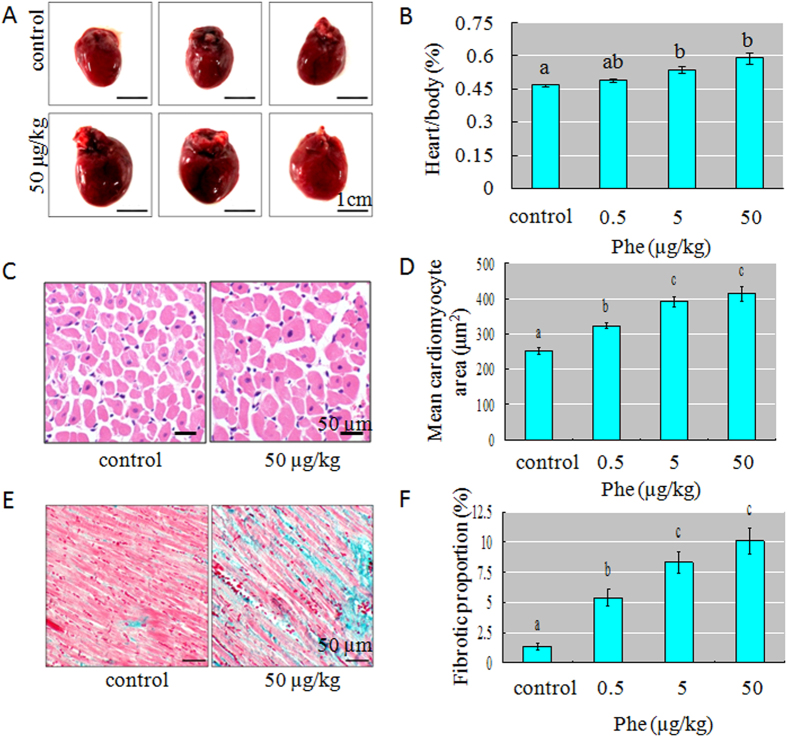
Phe induced cardiac hypertrophy in rats and H9 C2 cells. (**A**) Representative heart images showed that Phe exposure could induce cardiac hypertrophy in rats. Scale bar = 1 cm. (**B**) The heart weight to body weight (%) ratios (n = 10 per group) was significantly increased by Phe exposure. Means of exposures not sharing a common letter are significantly different at *P* < 0.05 (Dunnett). (**C**) Representative H&E staining images showed that Phe exposure could increase cardiomyocyte size in left ventricle sections of the rat heart. Scale bar = 50 μm. (**D**) The mean cardiomyocyte area quantified from images of sections was significantly increased by Phe exposure. Means of exposures not sharing a common letter are significantly different at *P* < 0.05 (Dunnett). (**E**) Representative Masson’s staining images showed that Phe exposure could increase the collagen deposited in the heart sections. Scale bar = 50 μm. (**F**) The area of cardiac fibrosis was significantly increased by Phe exposure. Means of exposures not sharing a common letter are significantly different at *P* < 0.05 (Dunnett).

In the present study, Phe exposed rats displayed cardiac hypertrophy (Fig. 1A). The heart weight to body weight (%) ratios was significantly increased after Phe exposure in a dose dependent manner (Fig. 1B).

In order to detect the histological changes, we performed sectioning and H&E staining. The results of mean cross-sectional area calculations showed that Phe increased cardiomyocyte size in the left ventricle (Fig. 1C,D).

Since myocardial fibrosis is a hallmark of hypertrophic cardiomyopathy, we detected the collagen deposition among the extracellular matrix using Masson’s staining. Our results showed that Phe increased the deposition of collagen in the heart sections (Fig. 1E,F).

Taken together, these results suggested that Phe could induce cardiac hypertrophy *in vivo*.

### Phe induced cardiac hypertrophy *in vitro*

**Figure 2:**
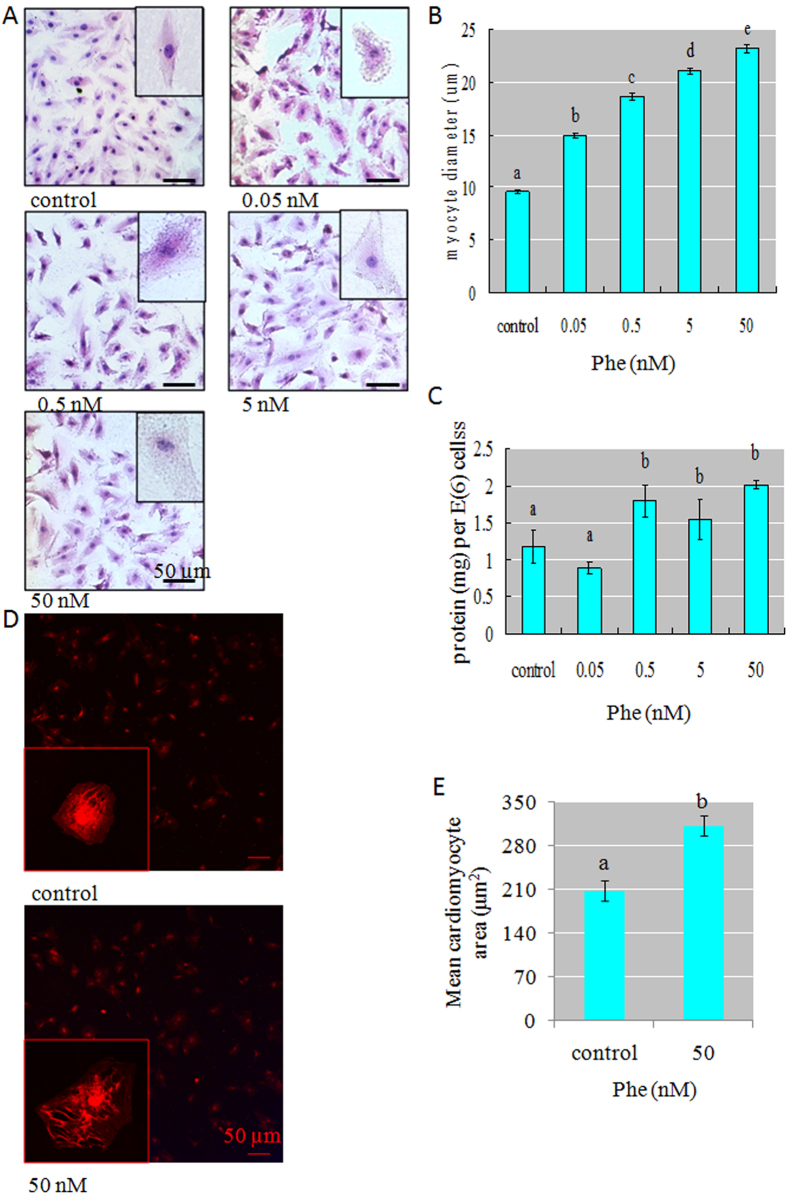
(**A**) Significant enlargement of the H9C2 cell size was visualized. Scale bar = 50 μm. (**B**) The average diameter measured from H9C2 cells was increased in the Phe-treated groups relative to untreated controls. Means of exposures not sharing a common letter are significantly different at *P* < 0.05 (Dunnett). (**C**) The normalized protein content in Phe-treated H9C2 cells was significantly increased, indicating an increase in protein synthesis. Means of exposures not sharing a common letter are significantly different at *P* < 0.05 (Dunnett). (**D**) Significant enlargement of the rat primary cardiomyocytes size was visualized. Scale bar = 50 μm. (**E**) The average area measured from rat primary cardiomyocytes was increased in the Phe-treated groups relative to untreated controls. Means of exposures not sharing a common letter are significantly different at *P* < 0.05 (Dunnett).

We further determined whether Phe could induce cardiac hypertrophy *in vitro*, using H9C2 cells and primary cardiomyocytes from rat. After exposure to a series of Phe concentrations for 24 h, a significant enlargement of the cell size was visualized in H9C2 cells (Fig. 2A). The average diameter of H9C2 cells increased more than 2-fold in the highest Phe-treatment relative to the untreated controls (Fig. 2B). To evaluate whether or not the increase in cell volume was caused by elevated organic cellular components or merely cell swelling, protein assay was performed, and the total protein content was normalized relative to the total cell numbers. The normalized protein content in Phe-treated cells was significantly increased up to 2-fold compared with that of the control, indicating an increase in protein synthesis (Fig. 2C). After exposure to 50 nM Phe for 24 h, a significant enlargement of the cell size was also visualized in primary cardiomyocytes from rat (Fig. 2D). The average area of H9C2 cells increased about 1.5-fold in the 50 nM Phe-treatment relative to the untreated controls (Fig. 2E). On the basis of this evidence, Phe was proved to be capable of inducing cardiac hypertrophy *in vitro*.

### Phe affected markers of cardiac hypertrophy

**Figure 3:**
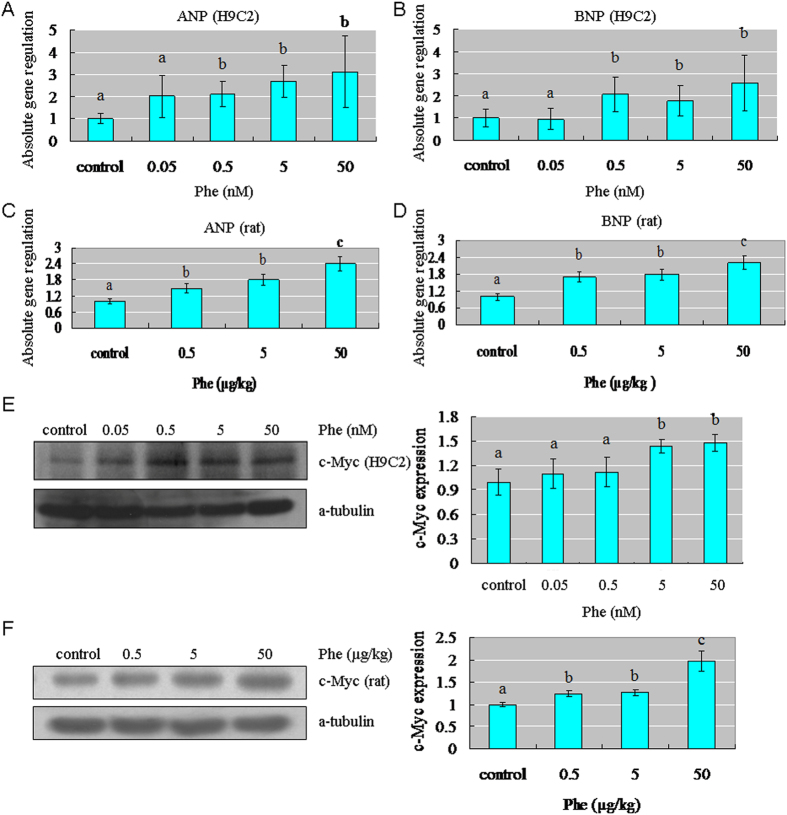
Phe affected markers of cardiac hypertrophy in H9 C2 cells and rat hearts. (**A–D**) Treatment with Phe effectively increased the mRNA levels of ANP and BNP in H9C2 cells and rat hearts. Means of exposures not sharing a common letter are significantly different at P < 0.05 (Dunnett). (**E,F**) After exposure to a series of Phe concentrations, a significant increase of the c-Myc expression was visualized in H9C2 cells and rat hearts. Intensities of c-Myc protein bands were quantified using densitometry. Results are expressed as multiples (×folds) of optical density of target protein and the alpha-tubulin determined in the control. The mean protein expression from the control was designated as 1 in the graph. Values (means ± S.D.) are representative of data obtained in three independent experiments (n = 3). Treatments not sharing a common letter are significantly different at P < 0.05 as assessed by one-way ANOVA followed by the Dunnett’s test.

We measured the mRNA levels of ANP and BNP, which are often used as markers of cardiac hypertrophy^31^,^32^. Treatment with Phe effectively increased the mRNA levels of these two genes in H9C2 cells and rat hearts (Fig. 3A–D). We also detected the protein expression level of c-Myc, which is another marker of cardiac hypertrophy^31^,^32^. Our results showed that, exposure to Phe significantly increased the c-Myc expression in H9C2 cells and rat hearts (Fig. 3E,F). These results further supported the notion that Phe could induce cardiac hypertrophy *in vitro*.

### Phe induced cardiac hypertrophy through reducing miR-133a, which then increased the level of CdC42 and RhoA

**Figure 4:**
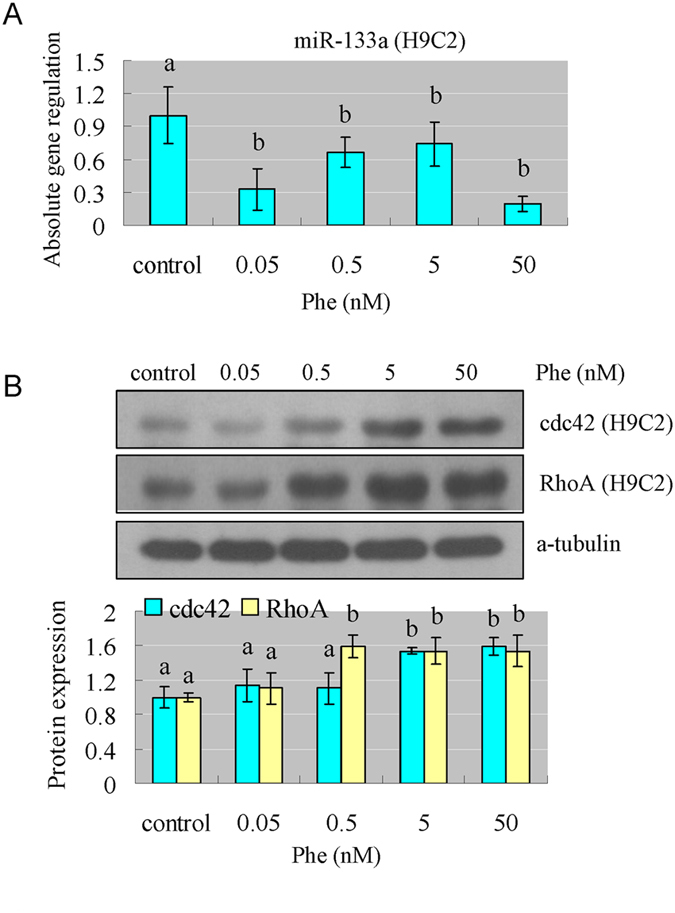
Phe affected miR-133a and transcription factors which are involved in the process of cardiac hypertrophy. (**A**) Phe exposure significantly reduced the level of miR-133a in H9C2 cells. Means of exposures not sharing a common letter are significantly different at P < 0.05 (Dunnett) (n = 6). (**B**) The protein expression level of CdC42 and RhoA, which are the specific target genes of miR-133a, were effectively increased after Phe exposure in H9C2 cells. Intensities of protein bands were quantified using densitometry. Results are expressed as multiples (×folds) of optical density of target protein and the alpha-tubulin determined in the control. The mean protein expression from the control was designated as 1 in the graph. Values (means ± S.D.) are representative of data obtained in three independent experiments (n = 3). Treatments not sharing a common letter are significantly different at P < 0.05 as assessed by one-way ANOVA followed by the Dunnett’s test.

**Figure 5:**
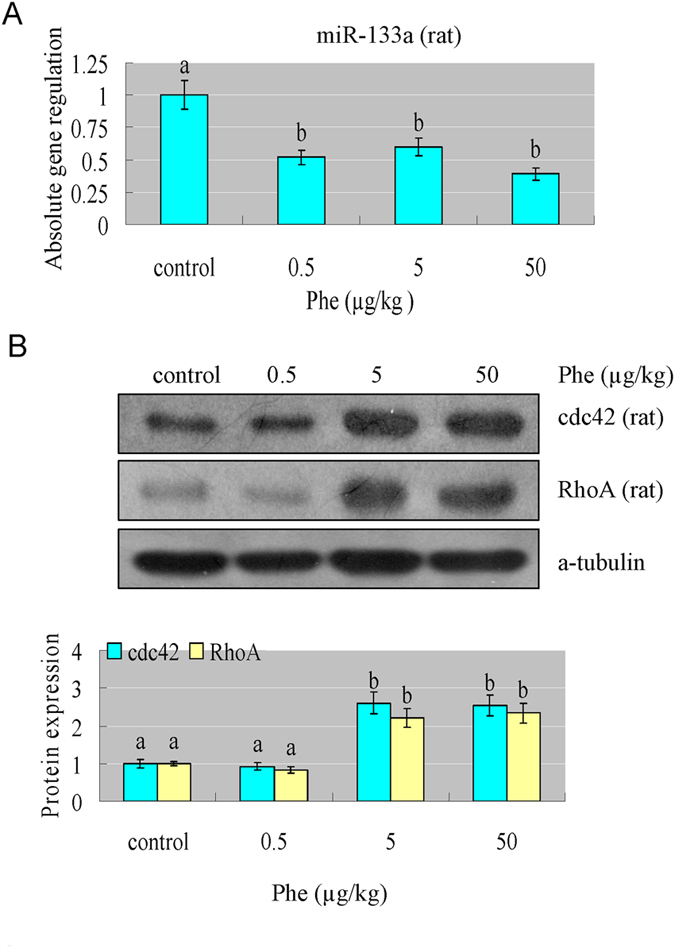
Phe affected miR-133a and transcription factors which are involved in the process of cardiac hypertrophy. (**A**) Phe exposure significantly reduced the level of miR-133a in rat hearts. Means of exposures not sharing a common letter are significantly different at P < 0.05 (Dunnett) (n = 6). (**B**) The protein expression level of CdC42 and RhoA, which are the specific target genes of miR-133a, were effectively increased after Phe exposure in rat hearts. Intensities of protein bands were quantified using densitometry. Results are expressed as multiples (×folds) of optical density of target protein and the alpha-tubulin determined in the control. The mean protein expression from the control was designated as 1 in the graph. Values (means ± S.D.) are representative of data obtained in three independent experiments (n = 3). Treatments not sharing a common letter are significantly different at P < 0.05 as assessed by one-way ANOVA followed by the Dunnett’s test.

As we know, miR-133a is a key regulator in determining cardiomyocyte hypertrophy^33^. We found that Phe exposure significantly reduced the level of miR-133a in H9C2 cells and rat hearts (Figs 4A and 5A). Since CdC42, RhoA and Nelf-A/WHSC2 are the specific target genes of miR-133a^33^, we also detected their protein expression levels. Our results showed that CdC42 and RhoA were effectively increased in H9C2 cells and rat hearts after Phe exposure (Figs 4B and 5B), while Nelf-A/WHSC2 was not significantly affected (data not shown). These results indicated that Phe might induce cardiac hypertrophy through reducing miR-133a levels, which then increased the levels of CdC42 and RhoA.

### Phe reduced miR-133a through increasing DNA methylation

**Figure 6:**
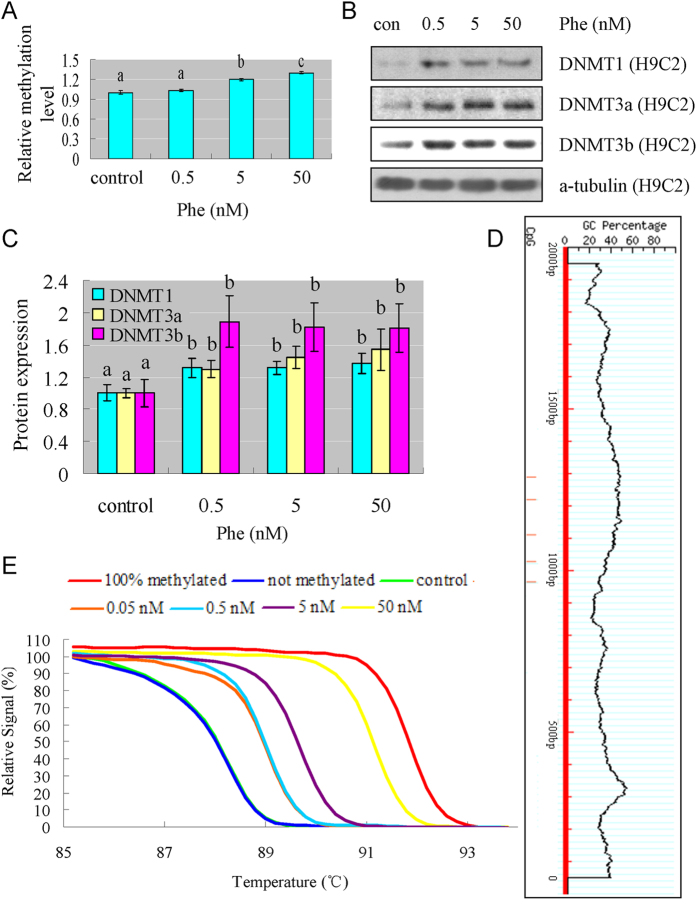
Phe increased global DNA methylation, expression of DNMT1, DNMT3a and DNMT3b, and the methylation level of the 5 CpG sites located at the putative transcription start sites of the miR-133a loci in H9C2 cells. (**A**) Phe increased global DNA methylation in a dose dependent manner. Means of exposures not sharing a common letter are significantly different at P < 0.05 (Dunnett). (**B,C**) Based on the results of Western blotting, we found that various doses of Phe were capable of increasing the expression of DNMT1, DNMT3a and DNMT3b in H9C2 cells. Intensities of protein bands were quantified using densitometry. Results are expressed as multiples (×folds) of optical density of target protein and the alpha-tubulin determined in the control. The mean protein expression from the control was designated as 1 in the graph. Values (means ± S.D.) are representative of data obtained in three independent experiments (n = 3). Treatments not sharing a common letter are significantly different at P < 0.05 as assessed by one-way ANOVA followed by the Dunnett’s test. (**D**) Schematic representation of the locations of the 5 CpG sites closely located at the putative transcription start sites of the miR-133a loci (−1000–−1300 bp) identified by the UCSC and MethPrimer database. Red lines indicate the positions of the CpG sites. (**E**) From the results of HRM assay, the melting curves of the control overlapped with the melting curves of the not methylated standard sample (blue). In Phe treated groups, variation in methylation was evident between not methylated and 100% methylated (red) standard sample. The position of the melting curves indicated that Phe exposure increased the methylation level of the 5 CpG sites located at the putative transcription start sites of the miR-133a loci.

One of the most important regulation mechanisms of miRNA transcription is DNA methylation. DNA hypermethylation can silence miR-133a^34^, and other research shows that Phe is capable of increasing DNA hypermethylation through DNMT1 and DNMT3b induction^35^. Since the reduction of miR-133a was observed in the present study, we examined whether the Phe-induced miR-133a reduction was caused by DNA methylation, and our results showed that Phe increased global DNA methylation in a dose dependent manner in H9C2 cells. Phe exposure at 50 nM for 12 hours increased the 5-methylcytosine content by 30% compared to control methylation (Fig. 6A).

**Figure 7:**
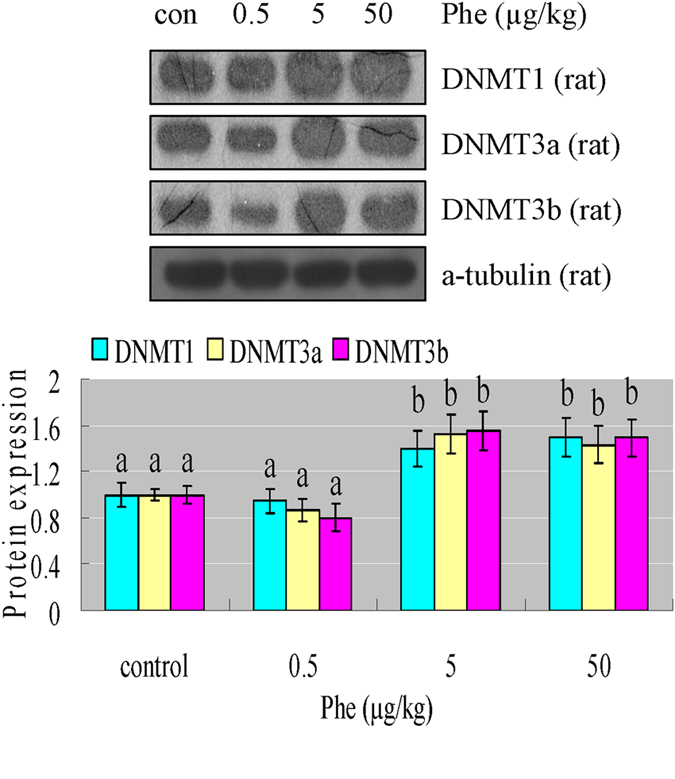
(**A**) Phe increased expression of DNMT1, DNMT3a and DNMT3b in rat hearts. Based on the results of Western blotting, we found that various doses of Phe were capable of increasing the expression of DNMT1, DNMT3a and DNMT3b in rat hearts. Intensities of protein bands were quantified using densitometry. Results are expressed as multiples (×folds) of optical density of target protein and the alpha-tubulin determined in the control. The mean protein expression from the control was designated as 1 in the graph. Values (means ± S.D.) are representative of data obtained in three independent experiments (n = 3). Treatments not sharing a common letter are significantly different at P < 0.05 as assessed by one-way ANOVA followed by the Dunnett’s test.

The DNA methyltransferase family of enzymes catalyze the transfer of a methyl group to DNA, and three active DNA methyltransferases have been identified in mammals: DNMT1, DNMT3a, and DNMT3b. From the Western blotting results, we found that various doses of Phe were capable of increasing the expression of DNMT1, DNMT3a and DNMT3b in H9C2 cells and rat hearts (Figs 6B,C and 7). This was a reasonable explanation for the increasing DNA methylation.

**Figure 8:**
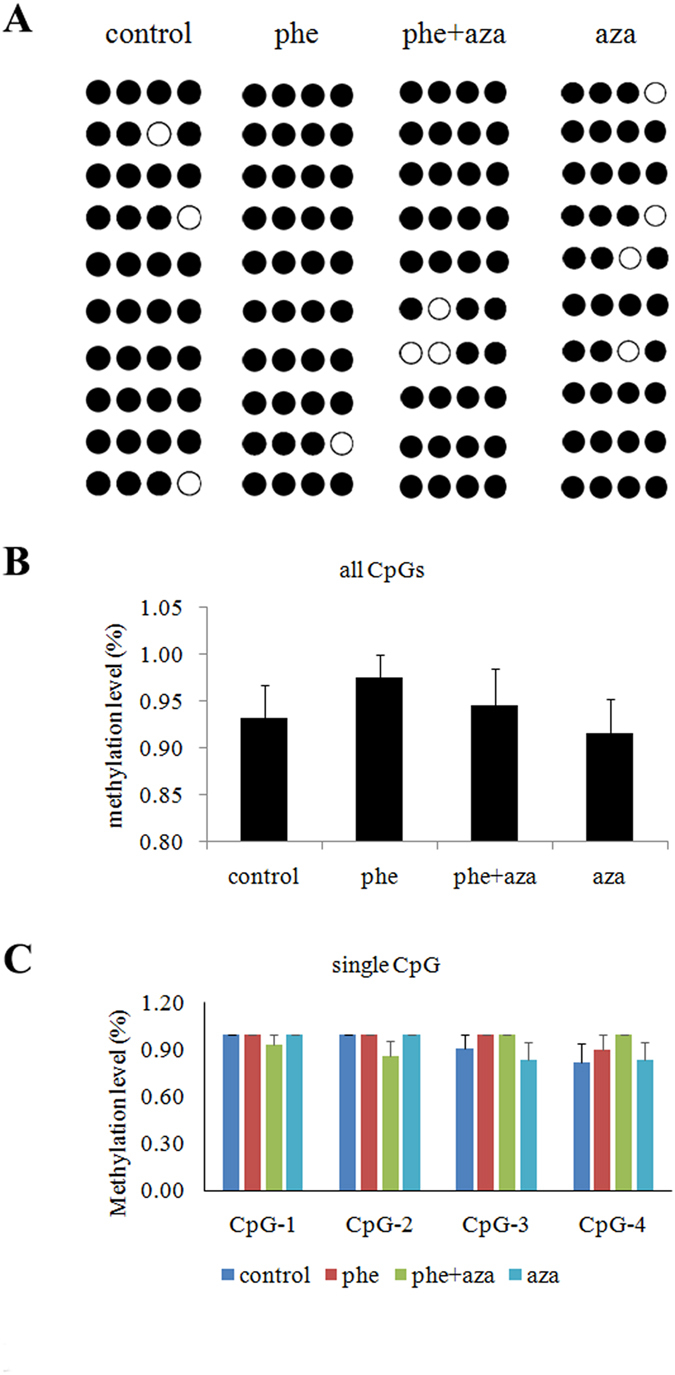
Methylation statuses of the CpG sites in Control, Phe treated group and DNMTs inhibitor treated group, as detected by bisulfite sequencing. (A). ○ represents unmethylated CpG sites; and ● represents methylated CpG sites. Each row represents a single sequence. The bar graphs depict the methylation rates of all 5 CpG sites (**B**) and single CpG sites (**C**).

Searches of the UCSC and MethPrimer database didn’t identify any CpG islands, but 5 CpG sites were found closely located at the putative transcription start sites of the miR-133a loci (−1000–−1300 bp) (Fig. 6D), which suggested that DNA methylation might control the transcriptional activity. Based on the results of HRM assay, the melting curves of the control overlapped with the melting curves of the not methylated standard sample. In Phe treated groups, variation in methylation was evident between the not methylated and 100% methylated standard samples. The position of the melting curves indicated that Phe exposure increased the accumulation of methylation level across the 5 CpG sites located at the putative transcription start sites of the miR-133a loci (Fig. 6E). Bisulfite sequencing was also performed to detect the methylation statuses of the 5 individual CpG sites (Fig. 8). Our results showed that the total frequency of methylation in Phe treated group was higher than the level measured in control group. Phe induced the methylation of CpG site 3 and 4, while the methylation level of CpG site 1 and 2 was not affected by Phe. Taking these results together, we think that Phe-induced miR-133a reduction was caused by DNA methylation.

**Figure 9:**
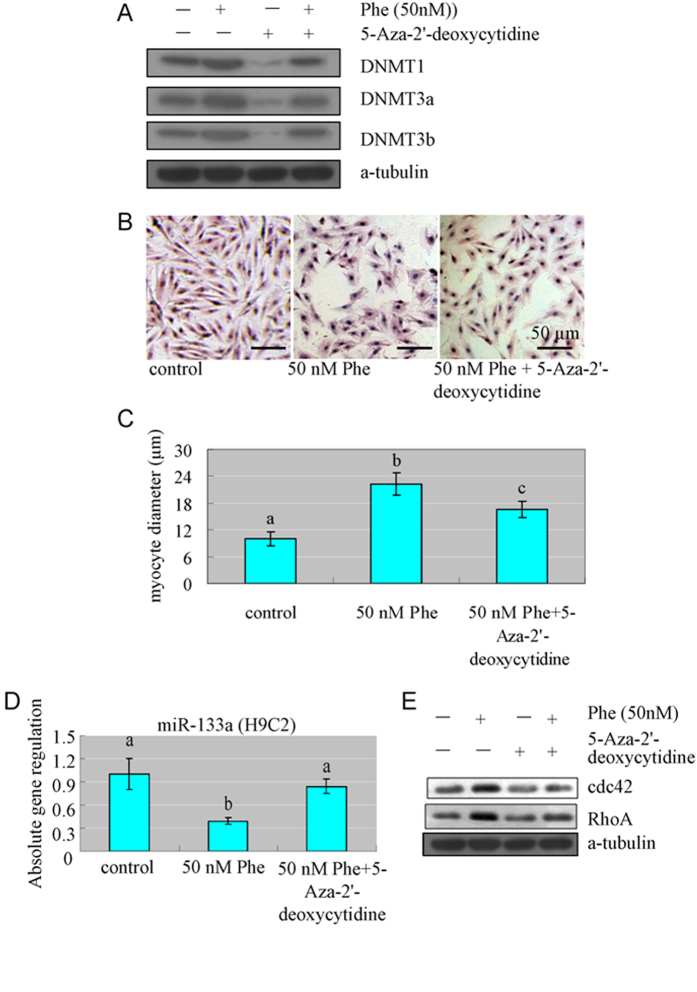
DNMTs inhibitor alleviated the enlargement of H9C2 cell size and perturbation of miR-133a, CdC42 and RhoA caused by Phe exposure. (**A**) DNMTs inhibitor alleviated the induction of DNMT1, DNMT3a and DNMT3b caused by Phe exposure. (**B,C**) When DNMTs were inhibited, the cell size reduced compared to the cells treated with Phe only. Means of exposures not sharing a common letter are significantly different at P < 0.05 (Dunnett). (**D,E**) When DNMTs were inhibited, the expression of miR-133a increased, while the expression of CdC42 and RhoA reduced, compared to Phe treated group. Means of exposures not sharing a common letter are significantly different at P < 0.05 (Dunnett).

In order to further determine the causal role of DNA methylation in the enlargement of cell size and perturbations of key regulators of cardiac hypertrophy, we exposed H9C2 cells to 50 nM Phe in the absence or presence of 10 μM DNA methylation inhibitor 5-aza-2′-deoxycytodine. Our results showed that, when DNMTs were inhibited (Fig. 9A), the cell size reduced compared to the cells treated with Phe only (Fig. 9B,C). At the same time, when DNMTs were inhibited, the expression of miR-133a increased, while the expression of CdC42 and RhoA reduced, compared to Phe treated group (Fig. 9D,E).

**Figure 10:**
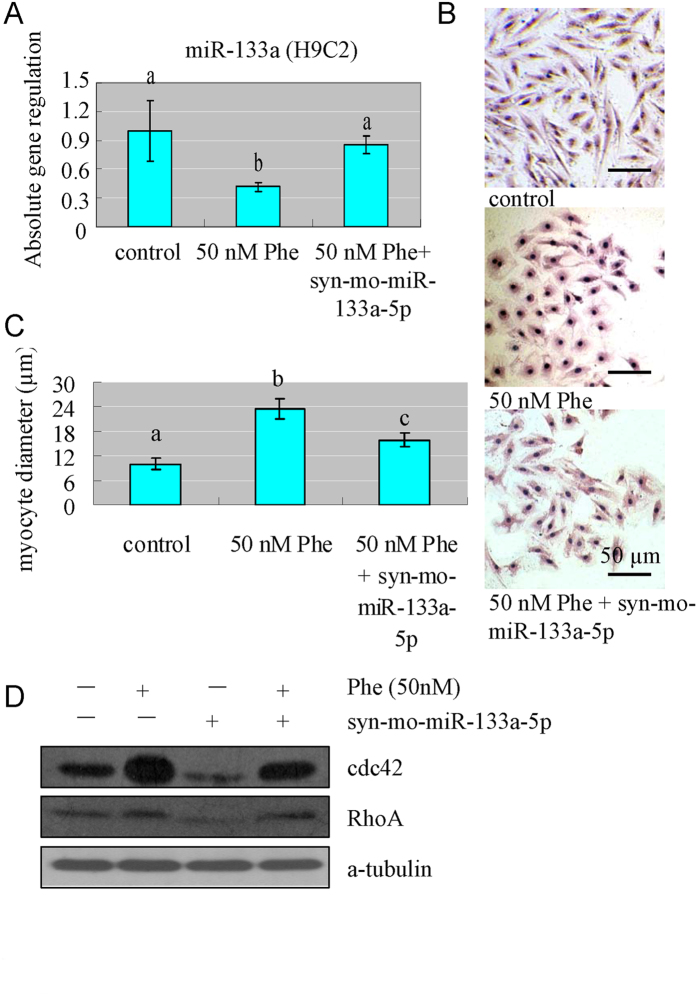
Overexpression of miR-133a alleviated the enlargement of H9C2 cell size and perturbation of CdC42 and RhoA caused by Phe exposure. (**A**) miR-133a was overexpressed by miR-133a mimics transfection. Means of exposures not sharing a common letter are significantly different at P < 0.05 (Dunnett). (**B,C**) When miR-133a was overexpressed, the cell size reduced compared to the cells treated with Phe only. Means of exposures not sharing a common letter are significantly different at P < 0.05 (Dunnett). (**D**) When miR-133a was overexpressed, the expression of CdC42 and RhoA reduced, compared to Phe treated group.

In order to further determine the causal role of miR-133a in the enlargement of cell size and perturbations of CdC42 and RhoA, we overexpressed miR-133a in H9C2 cells using miR-133a mimics in the absence or presence of 50 nM Phe. Our results showed that, when miR-133a was overexpressed (Fig. 10A), the cell size reduced compared to the cells treated with Phe only (Fig. 10B,C). At the same time, when miR-133a was overexpressed, the expression of CdC42 and RhoA reduced, compared to Phe treated group (Fig. 10D).

**Figure 11:**
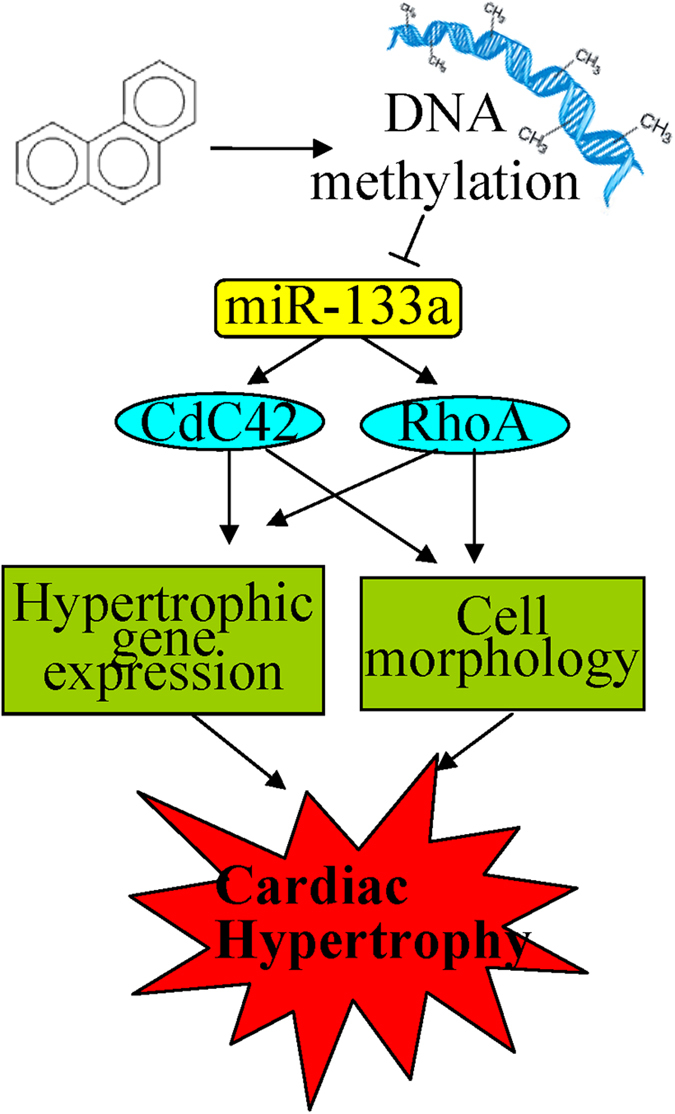
The overall scheme for Phe-induced cardiac hypertrophy.

In conclusion, Phe might induce cardiomyocyte hypertrophy through reducing miR-133a expression by DNA methylation, and the following reactivation of CdC42 and RhoA (Fig. 11).

## Discussion

Human exposure to PAHs may be continuous, where it exists in heavily polluted air, drinking water, smoked foods, and cigarette smoke. PAHs are proved to be cardiotoxic during early life development^26^,^27^,^36^. Recent research shows that embryonic exposure to very low dose of crude oil causes delayed physiological impacts on cardiovascular performance at later life stages^37^. Jules *et al.*^38^ found that in utero exposure to BaP predispose offspring to functional deficits in cardiovascular development that may contribute to cardiovascular dysfunction in later life. Weldy *et al.*^39^ proved that in utero and early life exposure to PAHs increases adult susceptibility to heart failure in mice. Several epidemiological, clinical, and *in vitro* studies linked the PAHs exposure to the development of cardiac hypertrophy^40^,^41^,^42^. Aboutab *et al.*^43^ demonstrated that BaP could induce the expression of the hypertrophic markers, atrial natriuretic peptide (ANP), and brain natriuretic peptide (BNP) and the heart to body weight ratio, reflecting the induction of cardiac hypertrophy in male SD rats. Therefore, environmental PAHs exposure during early life development might contribute to cardiovascular dysfunction in later life, including the cardiac hypertrophy, while the mechanism is not clear.

Our previous study shows that Phe exposure leads to an increase in intracellular Ca^2+^ concentration in H9C2 cells^29^. The results obtained in our present study demonstrated that Phe was capable of inducing the expression of ANP, BNP and c-Myc, accompanied by enlarged cell size, and increased protein synthesis, thereby resulting in cardiac hypertrophy. Since at the cellular level, cardiac hypertrophy is characterized by enlarged cell size, increased protein synthesis, an increase in intracellular Ca^2+^ concentration, and reactivation of cardiac hypertrophy markers such as the ANP, BNP, and c-Myc^31^,^32^, our results indicated that Phe exposure was capable of inducing cardiac hypertrophy.

As we know, miR-133a is demonstrated to have a critical role in determining cardiomyocyte hypertrophy by regulating its specific target genes: RhoA, CdC42, and Nelf-A/WHSC2^33^. From our results, Phe exposure reduced miR-133a, which then increased the level of CdC42 and RhoA, and the latter are implicated in the hypertrophic growth response, mediating both morphological changes and the changes in gene expression^44^.

It is reported that miR-133a can be silenced by DNA hypermethylation^34^, and that Phe is capable of increasing DNA hypermethylation through DNMT1 and DNMT3b induction^35^. Recent evidence has also shown that DNMT1, DNMT3a and DNMT3b may be targeted by PAHs, which then increase the DNA hypermethylation^45^,^46^,^47^. Since reduction of miR-133a was observed in the present study, we further evaluated the global DNA methylation level, the DNA methylation level of CpG sites within the miR-133a locus, and the expression of DNMT1, DNMT3a and DNMT3b. Our results showed that Phe was capable of increasing all of these. The results indicated that Phe might reduce miR-133a expression by increasing DNA methylation of CpG sites within the miR-133a locus.

In conclusion, we think that: 1) Phe could induce hypertrophy by perturbing defined miRNAs and transcription factors including miR-133a, CdC42 and RhoA (Fig. 9). These perturbations led to changes of protein synthesis, hypertrophic gene expression and cell size, thereby resulting in cardiac hypertrophy; and 2) Phe might reduce miR-133a expression by increasing the DNA methylation of CpG sites within the miR-133a locus (Fig. 9). Since the present study was conducted in ambient Phe concentrations, our results could have an important significance for preventing cardiac hypertrophy in humans.

Numerous studies have implicated miRNAs in cardiovascular physiology, as well as in the initiation and progression of cardiovascular diseases^48^. Their unique mode of action, fine-tuning gene expression rather than turning genes on/off, and their ability to simultaneously regulate multiple elements of relevant pathways makes them enticing potential biomarkers and therapeutic targets. The present study also showed that miRNAs might be potential biomarkers and therapeutic targets of cardiac diseases caused by environmental pollutions.

## Materials and Methods

### Animal experimental protocols

We confirm that all experiments were performed in accordance with relevant guidelines and regulations. All animal experiments were approved by the Animal Ethics Committee of Xiamen University (Acceptance No.: XMULAC20120030).

New born male Sprague-Dawley (SD) rats were purchased and housed in the Xiamen University Laboratory Animal Center (Xiamen University, Fujian, China) under institutional guidelines. Forty new born rats (5–6 g) without clinical signs were divided randomly into four experimental groups so that there were no statistically significant differences in body weights among groups. These rats received daily subcutaneous injections of Phe (Aldrich, Milwaukee, WI, USA) in corn oil (0.5, 5, 50 μg/kg), or corn oil vehicle alone (control), since postnatal days 0.5 (PND0.5). These concentrations were set according to the level of Phe detected in human subjects in our other research (3.5 ± 0.03 μg/kg). Individual animals were weighed before injection, and actual dosing volumes were adjusted based on the body weights recorded. The injection was continued until weaning (PND21), and then the rats were kept for another 3 weeks before being sacrificed (6 weeks old).

### Heart weight measurement

The hearts were dissected from the rats, washed in PBS to remove any blood, and placed on filter paper to remove the water. Then the hearts were weighed and normalized to body weight (n = 10).

### Histology

The hearts were fixed, embedded in paraffin, and cut in sections 4 μm in width and mounted on slides. For cardiomyocyte size analysis, sections were stained with hematoxylin and eosin (H&E). For cardiac fibrosis analysis, sections were stained with Masson’s staining according to the method modified from Sorriento (2010)^49^. Slides were stained with hematoxylin for 10 minutes, rinsed in PBS, and then stained with acid fuschin for 5 minutes. Slides were rinsed in PBS and stained with phosphomolybdic acid solution for 5 minutes, then stained with light green for 5 minutes. Slides were rinsed in distilled water, dehydrated with 95% and absolute alcohol, and a cover slip was placed. Sections were observed on a Nikon microscope, and imaged with a digital video camera. The mean cardiomyocyte area and the area of cardiac fibrosis was quantified from images of sections (n = 6) using IPwin software.

### Phe detection in the blood

The concentrations of Phe in the blood of control and 50 μg/kg Phe exposed group were measured using an Agilent 6890 gas chromatograph linked with an Agilent 5975B mass spectrometer (GC/MS) (Agilent Technologies, Palo Alto, CA, USA) (n = 3). Phe was extracted from the blood using dichloromethane. Clean up of the extracts was performed on 6% water deactivated aluminum oxide columns with hexane as the eluting solvent. The processed samples were analyzed for Phe using GC/MS in selected ion monitoring mode. Phe standards were prepared in hexane and used to develop a calibration curve. Phe was detected as an ion with a molecular weight of 178. The concentration of Phe in the blood of 50 μg/kg Phe exposed group was found to be 52.3 ± 5.8 nM.

### Cell culture

H9C2 rat cardiomyoblast cells were cultured at 37 °C with 5% CO2 in DMEM supplemented with 10% FBS, 1% penicillin/streptomycin, and 2 mM L-glutamine (Invitrogen, Carlsbad, CA, USA). The primary rat cardiomyocytes were cultured at 37 °C with 5% CO2 in a medium consisting of 4 parts DMEM, 1 part M199, 10% horse serum, 5% FBS, 1% penicillin/streptomycin. Phe was dissolved in DMSO and added to the medium at the indicated concentrations. The DMSO concentration in all treatments was 0.1%. Phe concentrations within 0.05–50 nM were chosen for cell exposure, since we found the concentration of Phe in the blood of 50 μg/kg Phe exposed group was 52.3 ± 5.8 nM. MTT assay also showed that 0.05–50 nM Phe had no inhibitory activity on H9C2 cells (data not shown). Therefore, cells were treated with 0.1% DMSO or 0.05–50 nM Phe, and collected for RNA/DNA extraction at 12 h, and for protein extraction at 24 h. There were six replicates for each of the treatments.

### Assessment of morphological change in cells

Medium was removed from the adherent H9C2 cell cultures followed by washing with PBS. H9C2 cells and primary rat cardiomyocytes were fixed in 2.5% formalin in PBS for 10 min, and then stained with H&E (Sigma Chemical Co.) and WGA^50^ (Invitrogen Co.), respectively. Morphological changes were visualized using a phase contrast microscope, and imaged with a digital video camera. The diameters of the minor axis of 100–120 cells from each group were assessed and recorded using IPwin software.

### Protein assay

Protein concentrations were measured using the Bradford method with a commercially available assay kit following the manufacturer’s instructions (Bio-Rad, Hercules, CA) and a Beckman DU 650 spectrophotometer (Pegasus Scientific, Frederick, MD). Protein content per cell was determined by dividing the total amount of protein by the cell number and was expressed as micrograms per 10^6^ cells (n = 6).

### QPCR analysis

QPCR analysis was performed as described in our previous research^51^, and gene expression levels of atrial natriuretic peptide (ANP) and B-type natriuretic peptide (BNP) were normalized to Rat GAPDH (glyceraldehyde-3-phosphate dehydrogenase) (n = 6). The following primers were used: 5′-GGT GCT GAG TAT GTC GTG GAG -3′ (GAPDH, forward), 5′-ACA GTC TTC TGA GTG GCA GTG AT -3′ (GAPDH, reverse), 5′-GCC CTG AGC GAG CAG ACC GA-3′ (ANP, forward), 5′-CGG AAG CTG TTG CAG CCT A-3′ (ANP, reverse), 5′-ACA ATC CAC GAT GCA GAA GCT -3′ (BNP, forward), 5′-CAG CTT GAA CTA TGT GCC ATC TTG -3′ (BNP, reverse).

### Western blotting assay

The total protein extracted from H9C2 cells and rat hearts (n = 3 per condition) with SDS-sample buffer were used for Western blotting with anti-alpha-tubulin, anti-c-Myc, anti-RhoA, anti-CdC42, anti-DNMT1, anti-DNMT3a, anti-DNMT3b antibody from Abcam (Cambridge, MA) based on our earlier research^52^. The protein band, specifically bound to the primary antibody, was detected using an anti-rabbit IgG-AP-linked antibody.

### Quantification of miR-133a

miR-133a was quantified using qRT-PCR TaqMan^®^ MicroRNA Assays (Applied Biosystems) in accordance with the manufacturer’s instructions, and normalized with U6 small nuclear RNA (snRNA) calculated by means of the 2^−ΔΔCt^ method^53^ (n = 6).

### Global DNA methylation measurement

Genomic DNA samples were treated with RNase A to remove RNA contaminants. The MethylampTM Global DNA Methylation Quantification Kit (Epigentek Group, Farmingdale, NY) was used to quantify the percentage of methylated cytosine in the genomic DNA following the manufacturer’s protocol (n = 6).

### Conversion of DNA bisulfite, methylation-specific high resolution melting (HRM) assay and bisulfite sequencing

Our study investigated the DNA methylation alterations to regions upstream of miR-133a. The UCSC and MethPrimer databases were used to acquire promoter regions upstream of miR-133a and to identify CpG islands based on other research^34^,^54^. Searches of the UCSC and MethPrimer database didn’t identify any CpG islands, but 5 CpG sites were found closely located at the putative transcription start sites of the miR-133a loci (−1000–−1300 bp), which suggested that DNA methylation might control the transcriptional activity.

Genomic DNA was subjected to bisulfite conversion using the EZ DNA Methylation-Gold kit (Zymo Research Corp., Orange, CA, USA). The conditions for the bisulfite conversion reaction were 98 °C for 10 min followed by 64 °C for 2.5 h, with a final incubation at 4 °C for up to 20 h in a PCR thermocycler. The modified genomic DNA was used for the HRM assay of the accumulation of methylation across the 5 CpG sites. PCR amplification and HRM analysis were carried out sequentially on a LightCylcer^®^ 480 (Roche, Penzberg, Germany). The following primers were used: 5′-AAT TTT GAG TAT TTG TTG AT-3′ (forward) and 5′-AAT CTA CAT CAA TTA TTA ATC ATT A-3′ (reverse). The amplification consisted of 10 min at 95 °C, followed by 50 cycles at 95 °C for 5 s, 58 °C for 5 s, and 72 °C for 5 s. Subsequently, the product was denatured for 1 min at 95 °C, re-annealed by fast cooling, and held for 1 min at 75 °C. The HRM analyses were performed in the temperature interval of 70–95 °C with 50 acquisitions/°C, and the default fluorescence temperature gradient parameters were selected. The melting curves were normalized by calculation of the ‘line of best fit’ between two normalization regions before and after the decrease in major fluorescence, which represented the melting of the PCR product using the software provided with LightCylcer^®^ 480. This algorithm allowed the direct comparison of samples that had different starting fluorescence levels.

Bisulfite sequencing was also performed. Modified DNA was amplified, and PCR products were gel-purified and sub-cloned into a pMD19-T vector system (TAKARA, Japan). Ten colonies were sequenced to assess the degree of methylation at each CpG site.

### Inhibition of DNA methylation

To determine the causal role of DNA methylation in the enlargement of cell size and perturbations of key regulators of cardiac hypertrophy, we exposed H9C2 cells to 50 nM Phe in the absence or presence of 10 μM DNA methylation inhibitor 5-aza-2′-deoxycytodine (Aldrich, Milwaukee, WI, USA).

### Over expression of miR-133a

To determine the causal role of miR-133a in the enlargement of cell size and perturbations of CdC42 and RhoA, we transfected miR-133a mimics (Qiagen, Venlo, Netherlands) into H9C2 cells in the absence or presence of 50 nM Phe, according to the manufacturer’s instructions.

### Data processing

Results were reported as means ± S.D. The data were statistically analyzed with one-way ANOVA followed by Dunnett’s multiple comparison tests via SPSS 13.0 software. A value of P < 0.05 was used to indicate significant difference.

## Additional Information

**How to cite this article**: Huang, L. *et al.* Phenanthrene exposure induces cardiac hypertrophy via reducing miR-133a expression by DNA methylation. *Sci. Rep.* **6**, 20105; doi: 10.1038/srep20105 (2016).
